# Early goal-directed therapy using a physiological holistic view: the ANDROMEDA-SHOCK—a randomized controlled trial

**DOI:** 10.1186/s13613-018-0398-2

**Published:** 2018-04-23

**Authors:** Glenn Hernández, Alexandre Biasi Cavalcanti, Gustavo Ospina-Tascón, Fernando Godinho Zampieri, Arnaldo Dubin, F. Javier Hurtado, Gilberto Friedman, Ricardo Castro, Leyla Alegría, Maurizio Cecconi, Jean-Louis Teboul, Jan Bakker, The ANDROMEDA-SHOCK Study Investigators, Glenn Hernandez, Gustavo Ospina-Tascón, Alexandre Cavalcanti, Arnaldo Dubin, Javier Hurtado, Gilberto Friedman, Ricardo Castro, Leyla Alegría, Jean-Louis Teboul, Maurizio Cecconi, Fernando Zampieri, Lucas Petri Damiani, Jan Bakker, Giorgio Ferri, Nicolás Rodriguez, Patricia Holger, Natalia Soto, Mario Pozo, Deborah Cook, Jean-Louis Vincent, Andrew Rhodes, Bryan Kavanagh, Phil Dellinger, Wim Rietdijk, David Carpio, Nicolás Pavéz, Elizabeth Henriquez, Sebastian Bravo, Emilio Daniel Valenzuela, Maria Alicia Cid, Ronald Pairumani, Macarena Larroulet, Edward Petruska, Claudio Sarabia, David Gallardo, Juan Eduardo Sanchez, Hugo González, José Miguel Arancibia, Alex Muñoz, Germán Ramirez, Florencia Aravena, Andrés Aquevedo, Fabián Zambrano, Milan Bozinovic, Felipe Valle, Manuel Ramirez, Victor Rossel, Pilar Muñoz, Carolina Ceballos, Christian Esveile, Cristian Carmona, Eva Candia, Daniela Mendoza, Aída Sanchez, Paula Fernández, Daniela Ponce, Jaime Lastra, Bárbara Nahuelpán, Fabrizio Fasce, Cecilia Luengo, Nicolas Medel, Cesar Cortés, Luz Campassi, Paolo Rubatto, Brenda Nahime Horna, Mariano Furche, Juan Carlos Pendino, Lisandro Bettini, Carlos Lovesio, María Cecilia González, Jésica Rodruguez, Elisa Estenssoro, Héctor Canales, Francisco Caminos, Cayetano Galletti, Estefanía Minoldo, María José Aramburu, Daniela Olmos, Nicolás Nin, Jordán Tenzi, Carlos Quiroga, Pablo Lacuesta, Agustín Gaudín, Richard Pais, Ana Silvestre, Germán Olivera, Gloria Rieppi, Dolores Berrutti, Marcelo Ochoa, Paul Cobos, Fernando Vintimilla, Vanessa Ramirez, Milton Tobar, Manuel Jibaja, Fernanda García, Fabricio Picoita, Nelson Remache, Vladimir Granda, Fernando Paredes, Eduardo Barzallo, Paul Garcés, Fausto Guerrero, Santiago Salazar, German Torres, Cristian Tana, José Calahorrano, Freddy Solis, Pedro Torres, Luís Herrera, Antonio Ornes, Verónica Peréz, Glenda Delgado, Alexei Carbonell, Eliana Espinosa, José Moreira, Diego Barahona, Blanca Salcedo, Ivonne Villacres, Jhonny Suing, Marco Lopez, Luis Gomez, Guillermo Toctaquiza, Mario Cadena Zapata, Milton Alonso Orazabal, Ruben Pardo Espejo, Jorge Jimenez, Alexander Calderón, Gustavo Paredes, José Luis Barberán, Tatiana Moya, Horacio Atehortua, Rodolfo Sabogal, Guillermo Ortiz, Antonio Lara, Fabio Sanchez, Alvaro Hernán Portilla, Humberto Dávila, Jorge Antonio Mora, Gustavo Ospina-Tascón, Luis Eduardo Calderón, Ingrid Alvarez, Elena Escobar, Alejandro Bejarano, Luis Alfonso Bustamante

**Affiliations:** 1 Departamento de Medicina Intensiva, Facultad de Medicina, Pontificia Universidad Católica de Chile, Diagonal Paraguay 362, Santiago, Chile; 2 Research Institute HCor, Hospital do Coração, R. Des. Eliseu Guilherme, 147 - Paraíso, São Paulo, Brazil; 3 Department of Intensive Care Medicine, Fundación Valle del Lili, Universidad ICESI, Carrera 98 # 18-49, Cali, Colombia; 4 Servicio de Terapia Intensiva, Sanatorio Otamendi y Miroli, Azcuénaga 894, Ciudad Autónoma de Buenos Aires, Argentina; 5 Centro de Tratamiento Intensivo, Hospital Español, Escuela de Medicina, Universidad de la República, Avda. Gral. Garibaldi, 1729 esq. Rocha, Montevideo, Uruguay; 6 Departamento de Medicina Interna, Faculdade de Medicina, Universidade Federal do Rio Grande do Sul, R. Ramiro Barcelos 2350 – Santa Cecilia, Porto Alegre, Brazil; 7 St George’s University Hospitals NHS Foundation Trust, Rd, London, SW17 0QT UK; 8 Service de Réanimation médicale, Hôpitaux universitaires Paris-Sud, Assistance Publique-Hôpitaux de Paris, Paris, France; 9 Division of Pulmonary, Allergy and Critical Care Medicine, Columbia University Medical Center, 630 W 168th St, New York, USA; 10 Department Intensive Care Adults, Erasmus MC University Medical Center, Rotterdam, CA The Netherlands; 11 Division of Pulmonary, and Critical Care Medicine, New York University-Langone, New York, USA

**Keywords:** Septic shock, Resuscitation, Peripheral perfusion, Lactate, Fluid responsiveness

## Abstract

**Background:**

Septic shock is a highly lethal condition. Early recognition of tissue hypoperfusion and its reversion are key factors for limiting progression to multiple organ dysfunction and death. Lactate-targeted resuscitation is the gold-standard under current guidelines, although it has several pitfalls including that non-hypoxic sources of lactate might predominate in an unknown proportion of patients. Peripheral perfusion-targeted resuscitation might provide a real-time response to increases in flow that could lead to a more timely decision to stop resuscitation, thus avoiding fluid overload and the risks of over-resuscitation. This article reports the rationale, study design and analysis plan of the ANDROMEDA-SHOCK Study.

**Methods:**

ANDROMEDA-SHOCK is a randomized controlled trial which aims to determine if a peripheral perfusion-targeted resuscitation is associated with lower 28-day mortality compared to a lactate-targeted resuscitation in patients with septic shock with less than 4 h of diagnosis. Both groups will be treated with the same sequential approach during the 8-hour study period pursuing normalization of capillary refill time versus normalization or a decrease of more than 20% of lactate every 2 h. The common protocol starts with fluid responsiveness assessment and fluid loading in responders, followed by a vasopressor and an inodilator test if necessary. The primary outcome is 28-day mortality, and the secondary outcomes are: free days of mechanical ventilation, renal replacement therapy and vasopressor support during the first 28 days after randomization; multiple organ dysfunction during the first 72 h after randomization; intensive care unit and hospital lengths of stay; and all-cause mortality at 90-day. A sample size of 422 patients was calculated to detect a 15% absolute reduction in mortality in the peripheral perfusion group with 90% power and two-tailed type I error of 5%. All analysis will follow the intention-to-treat principle.

**Conclusions:**

If peripheral perfusion-targeted resuscitation improves 28-day mortality, this could lead to simplified algorithms, assessing almost in real-time the reperfusion process, and pursuing more physiologically sound objectives. At the end, it might prevent the risk of over-resuscitation and lead to a better utilization of intensive care unit resources.

*Trial registration* ClinicalTrials.gov Identifier: NCT03078712 (registered retrospectively March 13th, 2017)

## Background

Septic shock is a potentially lethal condition associated with a mortality risk of up to 30–60% [1, 2]. Early recognition of tissue hypoperfusion and its reversion are key factors for limiting progression to multiple organ dysfunction and death [1–6].

Hyperlactatemia has been traditionally considered as a hallmark of ongoing tissue hypoxia and anaerobic metabolism [7, 8]. A recent study targeting a decrease in lactate levels as a resuscitation goal in critically ill patients showed a significant improvement in organ failure and outcomes associated with this endpoint [9]. Therefore, normalization of lactate levels has been recommended as a resuscitation target by current guidelines [10]. However, other non-hypoperfusion-related causes of hyperlactatemia might predominate in an unknown number of patients [11, 12]. In that setting, sustained efforts to increase cardiac output (CO) with fluids or vasoactive drugs could lead to detrimental effects of over-resuscitation [13, 14]. In addition, lactate exhibits a biphasic recovery rate even after a successful resuscitation [15], introducing an important confounder for practitioners.

Monitoring peripheral perfusion is particularly attractive because of its easy clinical accessibility and more importantly, because it could reflect the adequacy of intraabdominal visceral organ perfusion [16, 17]. The skin territory lacks auto-regulatory flow control, and therefore, sympathetic activation impairs skin perfusion during circulatory dysfunction [17], a phenomenon that could be evaluated by peripheral perfusion assessment. A robust body of evidence confirms that abnormal peripheral perfusion after initial resuscitation is associated with increased morbidity and mortality [18–23], whereby it could be used as a potential resuscitation goal [24]. In fact, the presence of a cold clammy skin, mottling or prolonged capillary refill time (CRT) has been suggested as indicators to initiate fluid resuscitation in patients with septic shock [17]. Interestingly, CRT was the first parameter to be normalized in patients surviving from septic shock and predicted lactate normalization at 24 h [18]. A recent pilot study suggests that targeting peripheral perfusion during septic shock resuscitation is safe and associated with less fluid administration and organ dysfunctions [25]. Therefore, the excellent prognosis associated with CRT recovery, its rapid-response time to fluid loading, its relative simplicity, its availability in resource-limited settings and its capacity to change in parallel with perfusion of physiologically relevant territories such as the hepatosplanchnic region [16] constitute strong reasons to evaluate the usefulness of CRT to guide resuscitation in septic shock patients.

Consequently, we decided to conduct a randomized controlled trial (RCT) comparing peripheral perfusion-targeted resuscitation (PPTR) versus lactate-targeted resuscitation (LTR) in patients with septic shock, hypothesizing that resuscitation aimed at peripheral perfusion will be associated with lower mortality rates. We also hypothesize that patients assigned to PPTR will require less volume of fluids with subsequent lower positive fluid balances. Accordingly, PPTR should be associated with less organ dysfunctions, especially at respiratory, renal and gastrointestinal levels.

## Methods

### Primary objective

To determine if PPTR is associated with lower mortality rates at 28 day than a LTR in patients with septic shock.

### Secondary objectives

To determine if a PPTR is associated with less severe multiple organ dysfunction; more mechanical ventilation (MV) free days; and less vasopressor load and renal replacement therapies (RRT) than a LTR strategy in patients with septic shock.

### Outcomes

Primary outcome will be all-cause mortality at 28-day.

Secondary outcomes:

- Free days of MV, RRT and vasopressor support during the first 28 days after randomization;
- Multiple organ dysfunction during the first 72 h after randomization [26];
- Intensive care unit (ICU) and hospital lengths of stay;
- All-cause mortality at 90-day.

Tertiary outcomes:

- Amount of resuscitation fluids at 8 and 24-hours;
- Total fluid balance at 8, 24, 48 and 72-h;
- Occurrence of intraabdominal hypertension (IAH) during the first 72 h after randomization (%);
- Use of RRT (%)
- In-hospital mortality

### Study design

ANDROMEDA-SHOCK is a prospective, multicenter, parallel-group, randomized trial aimed to compare an 8-h protocol of PPTR *vs.* LTR in patients with septic shock [27].

### Patients

Consecutive adult patients (≥ 18 years) with septic shock will be considered eligible. Septic shock is defined as suspected or confirmed infection, plus hyperlactatemia (≥ 2.0 mmol per liter) and vasopressor requirements due to refractory hypotension [27]. This latter is characterized as a systolic blood pressure (SBP) < 90 mmHg or a mean arterial pressure (MAP) < 65 mmHg after an intravenous fluid load of at least 20 ml/kg, administered over the course of 60 min.

Patients will be excluded in case of:

- pregnancy;
- anticipated surgery or dialysis procedure during the first 8 h after septic shock diagnosis;
- Do-Not-Attempt-Resuscitation status;
- active bleeding;
- acute hematological malignancy;
- concomitant severe acute respiratory distress syndrome (ARDS);
- more than 4 h after the onset of septic shock criteria.

An active daily screening for potentially eligible patients will be performed at all the participating ICUs.

### Randomization

**Fig. 1:**
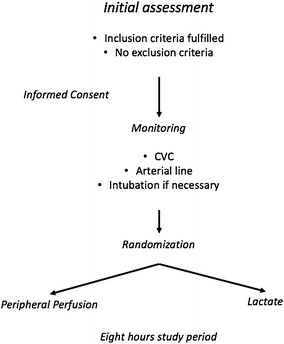
Pre-randomization phase assessments and interventions. *CVC* central venous catheter

Eligible patients will be randomly allocated to PPTR or LTR groups. PPTR will be aimed to normalize CRT, while LTR will target lactate normalization or a decreasing rate > 20% per 2 h of lactate levels during the 8 h of the study period (Fig. 1).

A randomization sequence with an allocation of 1:1 will be generated by a computer program. Study-group assignment will be performed by means of randomized permuted blocks of eight. Allocation concealment will be maintained by means of central randomization. Investigators at the sites will call a representative of the Study Coordinating Center (SCC) available 24 h/7 days through a dedicated phone number. The group to which the patient is allocated will only be disclosed after the information is recorded by the SCC. Such a measure prevents the investigator and the medical team from predicting to which treatment group the patient will be allocated.

## Interventions

### General management protocol

Both study groups will be treated with a common general management protocol. Sepsis source identification and control, and antimicrobial therapy will be given at the discretion of the treating physician. A central venous catheter (CVC) and an arterial line will be inserted in all cases, while the use of CO monitoring (pulmonary artery catheter or transpulmonary thermodilution techniques) is recommended for patients with a past medical history of heart failure or concomitant ARDS but leaving decision at discretion of the attending physician. Echocardiography will be performed routinely as soon as possible after admission to evaluate basal cardiac function, and to add in assessing fluid responsiveness (FR) [28]. Other dynamic predictors of response to fluids such as pulse pressure variation (PPV), stroke volume variation (SVV) or end-expiratory occlusion test (EEOT) will be used whenever applicable (see below) [28, 29]. MV will be provided (when needed) under light sedation (midazolam, propofol or dexmedetomidine) and analgesia (fentanyl, alfentanil, morphine); tidal volume (Vt) will be limited to 6–8 mL/kg and positive-end-expiratory-pressure (PEEP) will be set according to individual requirements [10]. Glycemic control will be adjusted to maintain glucose levels < 150 mg/dL. Norepinephrine (NE) will be the vasopressor of choice, and its dose will be adjusted to maintain a MAP ≥ 65 mmHg in all patients. Hemoglobin concentrations will be maintained at 8 g/dL or higher to optimize arterial O_2_ content. The use of other therapies such as epinephrine, vasopressin analogues, steroids or different blood purification techniques like high-volume hemofiltration (HVHF) will be provided according to the usual practice at the involved centers in patients evolving with refractory septic shock. Finally, stress ulcer and venous thrombosis prophylaxis will be managed according to international recommendations [10].

### Study protocol

**Fig. 2:**
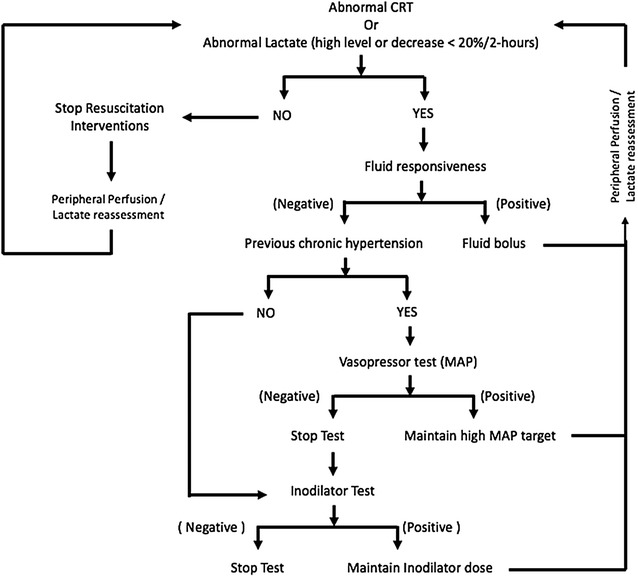
Resuscitation protocol for both groups. The figure describes the sequential approach to resuscitation. The process starts with fluid loading according to the status of fluid responsiveness. If the goal is not obtained, the second step is a vasopressor test, and then an inodilator test. *CRT,* capillary refill time

A sequential approach to resuscitation will be followed in both groups as shown in Fig. 2. After fulfilling inclusion criteria and discarding all exclusion conditions, an informed consent will be obtained. Basal measurements including hemodynamics and blood sampling will be performed at Time 0 (T0) representing the starting point just after randomization. The intervention period will be extended for 8 h. All other treatments, during the intervention period and after, will be at the discretion of the treating clinicians according to their local usual clinical practices.

## Tests and procedures during the study period

### Capillary refill time assessment

CRT will be measured by applying firm pressure to the ventral surface of the right index finger distal phalanx with a glass microscope slide. The pressure will be increased until the skin is blank and then maintained for 10 s. The time for return of the normal skin color will be registered with a chronometer. A CRT > 3 s will be considered as abnormal [30].

### Lactate measurements

A lactate value ≥ 2.0 mmol per liter will be considered as abnormal. Arterial lactate levels will be measured at each center, either at point of care or central laboratories (point of care: GEM 4000, Instrumentation Lab, IL, USA; central laboratories: Cobas b221, Roche Diagnostics International; Basel, CH).

### Fluid responsiveness (Fig. 3)

**Fig. 3:**
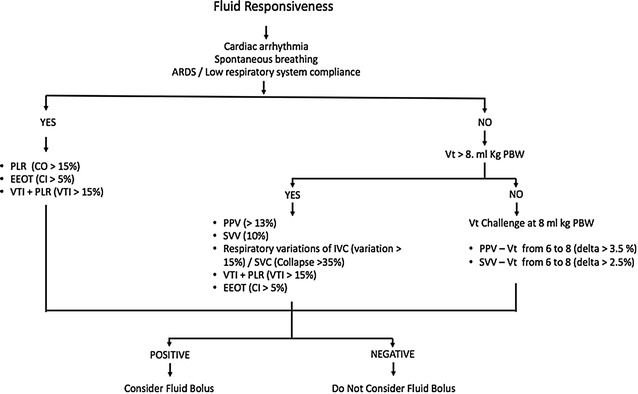
Assessment of fluid responsiveness during the study period. The figure describes an algorithm for assessing fluid responsiveness in different settings depending on the presence or not of mechanical ventilation, arrhythmias or other conditions. Different tests are proposed with the respective cutoff values. *ARDS* acute respiratory distress syndrome; *PLR* passive leg rising; *CO* cardiac output; *EEOT* end-expiratory occlusion test; *CI* cardiac index; *VTI* velocity time integral; *Vt* tidal volume, *PBW* predicted body weight; *PPV* pulse pressure variation; *SVV* stroke volume variation, *IVC* inferior vena cava; *SVC* superior vena cava

FR will be assessed using a structured approach outlined in Fig. 2. Dynamic predictors of FR will be evaluated depending on the individual status, i.e., considering if under MV or spontaneous breathing, Vt, respiratory rate (RR), respiratory system compliance and the presence of arrhythmias. The protocol for patients under MV is shown in Fig. 3 [28, 29].

### Fluid challenges

In FR^+^ patients, the first resuscitation step will be to administer a fluid bolus (FB) of 500 ml of crystalloids every 30 min until normalizing CRT in the PPTR group. In the LTR group, FB will be stopped if at 2 h lactate is normalized or has decreased > 20%, or previously if after any of the fluid boluses, central venous pressure (CVP) has increased ≥ 5 mmHg or the patients have become fluid unresponsive (FR^−^).

### Safety measures during fluid challenges

CVP and FR will be reevaluated after any fluid challenge. If CVP increases < 5 mmHg and FR is still +, another FB will be administered and so on while the perfusion (CRT or lactate) goal are not attained. If CVP increases ≥ 5 mmHg or FR is or become negative, fluids will be stopped and the patient will be moved to the next step.

### Vasopressor test

An open-label vasopressor test will be performed increasing MAP up to 80–85 mmHg by using progressive incremental doses of NE in patients with previous history of chronic hypertension and persistently abnormal CRT or unfulfilled lactate goals accompanied by a fluid unresponsive state. Parameters will be reassessed 1 h after in the PPTR and 2 h after in the LTR. If after the vasopressor test, CRT improves, and lactate goals are achieved in PPTR and LTR, respectively, NE will be titrated to maintain this new MAP goal throughout the study period. If goals are not achieved despite increasing MAP, or NE dose surpasses 0.8 mcg/kg/min or adverse effects are observed (heart rate (HR) > 140 ppm, arrhythmias or evident cardiac ischemia), NE dose will be reduced to the level before the vasopressor test, and the protocol will move to the next step.

### Inodilator test

An open-label test of dobutamine at fixed 5 mcg/kg/min or milrinone at fixed 0.25 mcg/kg/min doses (at discretion of the attending physician) will be started in patients with persistent abnormal CRT or non-achieved lactate goals, and negative FR status. CRT and lactate goals will be rechecked such as in the vasopressor test. If such resuscitation goals are not reached, drugs will be discontinued and no further action will be taken during the study period, except for rechecking FR every hour and restart fluid challenges if the patient gets again FR + . Dobutamine or milrinone doses will be maintained throughout the study period in those favorably responding to the open-label inodilators test. As a safety measure, inodilators will be stopped if HR increases > 15%, or arrhythmias, ischemia or hypotension develop.

## Management of peripheral perfusion-targeted resuscitation

As a safety measure, if signs of inadequate macrohemodynamics persist such as HR > 120 BPM or unstable MAP with increases in vasopressors during the last hour, resuscitation will be continued even if CRT is normal.

After CRT normalization at any step, it will be reassessed hourly during the study period. If at any point it turns abnormal again, the resuscitation sequence will be restarted (Fig. 2)

## Management of lactate-targeted resuscitation

Lactate will be assessed every 2 h during the 8-h study period. If after achieving lactate goals, it becomes again abnormal or the decrease rate slow down under 20%/2 h at any of the following controls, the resuscitation sequence will be restarted (Fig. 2).

### Safety measures

The protocol can be stopped at any moment for safety considerations during the 8-h study period if the attending intensivist considers that the patient has developed unexpected and severe complications or evolves into refractory shock, conditions that under his judgment require liberalization of management. This action has to be reported on the case report form (CRF), and the patient will be followed up with major outcomes, and included in the intention-to-treat (ITT) analysis. Specific safety measures for fluid administration, vasopressor test and inodilator use are specified above.

### Suspected unexpected serious adverse reactions (SUSAR)

Any adverse event that occurs in a clinical trial subject, which is assessed by the study investigator as being unexpected, serious and as having a reasonable possibility of a causal relationship with the study procedure will be reported. Reports of these reactions are subject to expedited submission to health authorities. SUSAR’s will be analyzed by both the SCC and the data safety monitoring committee (DSMC).

### Blinding

Since the intervention will be administered to critically ill patients (mostly sedated), blinding of these patients is not necessary. Because this is a non-pharmacological intervention, blinding of the medical team is not feasible.

### Data collection and management

Study follow-up and the variables that will be collected are described below.

### Baseline

Demographics, comorbidities, acute physiology and chronic health evaluation (APACHE) II [31], sepsis source and treatment

pre-ICU resuscitation and fluid balance

Sequential Organ Failure Assessment (SOFA) [26] + and Acute Kidney Injury Network (AKI) criteria [32]

*Hemodynamics*: HR, SBP, diastolic blood pressure (DBP), MAP, CVP, FR status, intraabdominal pressure (IAP), NE levels, diuresis.

*Perfusion*: lactate, central venous O_2_ saturation (ScvO_2_), central venous arterial pCO_2_ gradient (P(cv-a)CO_2_), Hb, central venous and arterial blood gases, CRT, mottling score.

### Evolution

- SOFA and AKI criteria at 8, 24, 48 and 72 h
- Hemodynamics hourly up to 8 h
- Fluid administration and balance at 8, 24, 48 and 72 h
- Complete perfusion assessment when the targeted parameter is normalized and then at 8, 24, 48 and 72 h
- Register of vasoactive drugs and dobutamine/milrinone use
- Register of MV and RRT
- Source control re-analysis at 4 h
- Rescue therapies: HVHF, vasopressin, epinephrine, steroids, others.
- Echocardiography at least once during the study period
- Follow-up till 28 days for use of MV, RRT and vasopressors
- All-cause mortality at hospital discharge, 28 and 90 days
- Cause of death.

### Quality control

Several procedures will assure data quality, including (1) all investigators will attend a training session before the start of the study to standardize procedures, including data collection (2) the investigators may contact the SCC to solve issues or problems that may arise; (3) CRFs provided by the centers will be subjected to various checks by members of the SCC for missing data, plausible, possible or non-permitted value ranges, and logic checks on a weekly basis. (4) centers will be notified of the inconsistencies or missing data as queries and asked to correct them; (5) the SCC will review detailed reports on screening, enrollment, follow-up, inconsistencies and completeness of data. Immediate actions will follow to solve problems that arise; (6) only after the CRFs are cleared by the SCC, data will be entered in the final electronic database by the data digitizer.

### Sample size

Mortality in patients with increased lactate levels in circulatory dysfunction has been shown to exceed 40% [9]. In addition, several studies have shown that abnormal peripheral perfusion is associated with a mortality exceeding 40% as well, whereas a normal CRT in the early phase of septic shock has been associated with a less than 10% mortality [19, 30].

A total sample size of 420 patients (210 per group), analyzing the data using the ITT principle, is expected to provide approximately 90% power to detect a reduction in 28-day mortality from 45 to 30%, considering logistic regression, with a two-sided alpha level of 5%. We consider a decrease of 15% in mortality to have direct clinical implementation effect. Similar effects on mortality have been shown in early resuscitation studies. In addition, limiting fluid administration in patients with septic shock and normal peripheral perfusion has been shown to decrease organ failure, which is the leading cause of death in these patients [25].

However, if a smaller decrease in mortality (such as 10%) is observed at interim analyses, our initial calculated sample size would have only a 57% power to detect benefit. Therefore, we will use an adaptive approach [33] that will allow for a sample-size re-estimation at a preplanned interim analysis after 75% of the sample has been recruited. The sample-size re-estimation will be conducted by the DSMC only if the size effect observed in the interim analysis is between 10 and 15% absolute reduction in mortality [32].

### Statistical analysis plan

We will report a detailed statistical analysis plan in a separate document.

Briefly, all analysis will follow the intention-to-treat principle.

### Primary outcome

We will assess the effect of PPTR compared to LTR on the primary outcome using time-to-event analysis. Results of our main analysis will be calculated with Cox proportional hazards models, with adjustment for five pre-specified baseline covariates. APACHE II score, SOFA score, lactate level, CRT and source of infection, as fixed (individual-level) effects. Results will be reported as hazard ratios with 95% confidence intervals (CI) and *p* values. We will also present Kaplan–Meier curves.

### Secondary outcomes

We identified several secondary outcomes. First, binary outcomes will be compared through Chi-squared tests, and we will present the results risk ratios (RR), with 95% CI and *p* values.

Continuous outcomes with normal distribution will be analyzed with *t* test and reported as mean difference 95% CI and *p* value. Continuous outcomes with asymmetrical distribution will be analyzed using bootstrapping techniques and reported as absolute difference between medians, 95% CI and *p* values.

### Subgroup analyses

We will analyze the effects of resuscitation strategies on the primary outcome in the following subgroups:

- Patients with lactate > 4.0 mmol/L as set by SSC [10]
- Patients without a confirmed source of infection (as this could increase the translation of the study to other critically ill).
- Patients with low APACHE II/SOFA scores
- Patients with a more than 10% difference in lactate levels between the very first one measured and the baseline when starting the study.

### Ethical aspects

Each investigator center will submit the study protocol to its Institutional Review Board (IRB). The study will start only after being approved by the IRB. Written informed consent will be obtained from a legal representative of all participants. This study is in compliance with local and international declarations.

### Trial organization and management

#### Study Coordinating Center

A team based on the Departmento de Medicina Intensiva, Facultad de Medicina of Pontificia Universidad Católica, Chile, will manage the trial on a day-to-day basis. The SCC is comprised by the chief and co-chair investigators, four project managers, a statistician and a data digitizer. The statistician is based on the Research Institute HCor, São Paulo, Brazil.

The responsibilities of the SCC include:

1. *Planning and conducting the study* designing the protocol; designing the CRF; designing the operation guide; managing and controlling data quality; designing, testing and maintaining the electronic database; data quality control; assisting the steering committee;
2. *Managing the research centers* selecting and training the research centers; helping the centers prepare a regulatory report to be submitted to the IRBs and assisting the centers with the submission; monitoring recruitment rates and the actions to increase recruitment; monitoring follow-up and implementing actions to prevent follow-up losses; auditing; sending study materials to the research centers; producing a monthly study newsletter; developing supporting material for the study;
3. *Statistical analysis and research reporting* complete statistical analysis; helping to write the final manuscript.

### Trial Steering Committee

The Trial Steering Committee (TSC) is responsible for the overall study supervision, assisting in developing the study protocol and preparing the final manuscript. All other study committees report to the TSC. The TSC members are investigators trained in designing and conducting randomized clinical trials in critically ill patients.

### Study centers

The study centers for ANDROMEDA-SHOCK were selected through a rigorous process. This started with a survey of professional and technical resources as well as processes of care. Centers were contacted trying to make this process representative across public, private and university hospitals, different countries and cultures, and hospital size.

At the end, 34 centers were selected and all applied for IRB approval, leaving finally 26 active centers to start on March 1, 2017, in 5 countries. Brazil is still pending

Details of the centers which accepted to participating in the trial at the time of this manuscript submission are given in the Appendix.

### Publication policy

The ANDROMEDA-SHOCK study success depends on all its collaborators. Therefore, the primary results of the trial will be published under the name of ANDROMEDA-SHOCK Investigators. The contributions of all collaborators, their names and respective institutions, will be acknowledged in the manuscript. To safeguard the scientific integrity of the study, data from this study will be submitted to publication only after the final approval from the TSC.

### Data Safety Monitoring Committee

The DSMC is set up with independent epidemiologists and intensivists. The DSMC is in charge of providing recommendations for the SCC of continuing the study as planned or discontinuing the recruitment based on evidence that the intervention causes increased mortality in the experimental group (PPTR) as compared to the control group (LTR). Interim analyses will be conducted after recruitment of the first 100 patients and at 75% of the sample.

In addition, the DSMC will discuss and potentially recommend a re-estimation of the sample size according to the interim analysis after recruitment of 75% of the patients. A sample-size re-estimation design is a flexible, adaptive design with the primary purpose of allowing sample size of a study to be reassessed in the mid-course of the study to ensure adequate power.

## Discussion

ANDROMEDA-SHOCK is a relevant study in septic shock for several reasons: (1) it determines the value of a simple, bedside, universally available parameter to be used as a resuscitation goal in early septic shock; (2) it proposes an early goal-directed resuscitation strategy based on a holistic physiological view of the reperfusion process; (3) it challenges the gold-standard parameter of lactate since this latter is not universally available and has many interpretation difficulties.

If our hypothesis proves to be correct, resuscitation algorithms might be simplified, assessing almost in real-time the reperfusion process, and in pursuing more physiologically sound objectives through a peripheral perfusion-based strategy, it could prevent the risk of over-resuscitation and lead to a better utilization of ICU resources.

### Study status

ANDROMEDA-SHOCK study started recruiting on March 1 in 26 centers from five countries. At the submission of this manuscript, already 388 patients have been recruited.

## Abbreviations

CO: cardiac output
CRT: capillary refill time
RCT: randomized controlled trial
PPTR: peripheral perfusion-targeted resuscitation
LTR: lactate-targeted resuscitation
MV: mechanical ventilation
RRT: renal replacement therapy
ICU: intensive care unit
IAH: intraabdominal hypertension
SBP: systolic blood pressure
MAP: mean arterial pressure
SCC: Study Coordinating Center
CVC: central venous catheter
FR: fluid responsiveness
PPV: pulse pressure variation
SVV: stroke volume variation
EEOT: end-expiratory occlusion test
Vt: tidal volume
PEEP: positive-end-expiratory-pressure
NE: norepinephrine
HVHF: high-volume hemofiltration
RR: respiratory rate
VTI: aortic velocity time integral
PLR: passive leg raising
HR: heart rate
CRF: case report form
ITT: intention-to-treat
DSMC: data safety monitoring committee
APACHE: acute physiology and chronic health evaluation
AKI: acute kidney injury network
DBP: diastolic blood pressure
SUSAR: suspected unexpected serious adverse reactions
IAP: intraabdominal pressure
ScvO_2_: central venous oxygen saturation
P(cv‐a) CO_2_: central venous‐arterial PCO_2_ gradient
IRB: Institutional Review Board
TSC: Trial Steering Committee
